# Extracellular Vesicle lncRNA Metastasis-Associated Lung Adenocarcinoma Transcript 1 Released From Glioma Stem Cells Modulates the Inflammatory Response of Microglia After Lipopolysaccharide Stimulation Through Regulating miR-129-5p/High Mobility Group Box-1 Protein Axis

**DOI:** 10.3389/fimmu.2019.03161

**Published:** 2020-02-07

**Authors:** Jiankai Yang, Guozhu Sun, Yuhua Hu, Jipeng Yang, Yijun Shi, Hongjiang Liu, Chen Li, Yuanyu Wang, Zhongqiang Lv, Jianxing Niu, Honglei Liu, Xuefang Shi, Haiping Wang, Pan Li, Baohua Jiao

**Affiliations:** ^1^Department of Neurosurgery, The Second Hospital of Hebei Medical University, Shijiazhuang, China; ^2^Laboratory Diagnosis Center, Beijing Tiantan Hospital, Capital Medical University, Beijing, China; ^3^Department of Neurosurgery, The Third Medical Center of Chinese PLA General Hospital, Beijing, China; ^4^Department of Neurosurgery, Shijiazhuang Third Hospital, Shijiazhuang, Hebei, China; ^5^International Department, Children's Hospital of Hebei Province, Shijiazhuang, China

**Keywords:** extracellular vesicles, metastasis-associated lung adenocarcinoma transcript 1 (MALAT1), miR-129-5p, high mobility group box-1 protein (HMGB1), microglia

## Abstract

Glioma stem cell (GSC)–derived extracellular vesicles (EVs) can mediate the communication between GSCs and microglia. Metastasis-associated lung adenocarcinoma transcript 1 (MALAT1) expression in GSCs, EVs, and supernatant was detected by real-time PCR. The direct targeting between MALAT1 and miR-129-5p, miR-129-5p, and HMGB1 were tested with luciferase reporter analysis. The expression and secretion of interleukin (IL)-6, IL-8, and tumor necrosis factor (TNF)-α were determined in lipopolysaccharide-stimulated microglia or miR-129-5p inhibitor transferred to microglia exposed to GSC EVs or EVs derived from siMALAT1 pre-transferred GSCs. MALAT1 was enriched in GSC EVs compared with GSCs, and up-regulated MALAT1 was also observed in microglia upon GSC EVs incubation. The relative expression and secretion of IL-6, IL-8, and TNF-α in lipopolysaccharide-stimulated microglia were up-regulated in the GSC supernatant group, which could be reversed by dimethyl amiloride (DMA) (EV secretion inhibitor) co-administration or si-MALAT1 pre-transfection of GSCs. Luciferase reporter assay testified the direct binding of MALAT1 and miR-129-5p, miR-129-5p, and HMGB1, and si-MALAT1 could up-regulate miR-129-5p expression and down-regulate HMGB1 expression in microglia cells. The concentration of IL-6, IL-8, and TNF-α in lipopolysaccharide-stimulated microglia exposed to EVs from siMALAT1 transfected GSCs could be up-regulated by miR-129-5p inhibition. EVs lncRNA MALAT1 released from GSCs could modulate the inflammatory response of microglia after lipopolysaccharide stimulation through regulating the miR-129-5p/HMGB1 axis.

## Introduction

Originated from brain or spine glial cells, glioma may account for nearly 80% of malignant cerebral tumors. According to the WHO Neuropathological Classification of Tumors of the Central Nervous System, glioma can be classified into low-grade gliomas (WHO grade II) and high-grade (WHO grade III–IV). The ineffectiveness of the treatments for glioma, especial high-grade glioma, majorly results from the multipotent tumor-initiating glioma stem cells (GSCs), which have been indicated to participate in tumor aggressiveness and radio- or chemoresistance. It has been further testified that major histocompatibility complex and o-stimulatory molecules are down-regulated on GSCs, while immune-inhibitory molecules are up-regulated (1). Thus, further understanding of the immunosuppression mechanism relevant to GSCs will be necessary for developing efficient therapeutic strategies (2, 3).

Macrophages and/or microglia may account for a considerable portion of glioma mass (4), and the number and intensity of the infiltrated microglia/macrophages are intimately correlated with the metastasis and progression of glioma. Substantial evidence indicates the indispensable role of the immune-suppressive milieu induced by the interaction between glioma and microglia/macrophages, which will eventually favor the growth, invasion, metastasis, and neoangiogenesis of glioma cell (5). However, the precise functioning and underlying mechanism involved in the interaction of GSCs and tumor-infiltrated microglia are still to be addressed.

Extracellular vesicles (EVs), newly identified lipid bilayer–delimited particles of endocytic origin (30–100 nm), can convey cargoes such as signal peptides, lipids, mRNA, microRNA, and lncRNA to distant and nearby cells as intercellular communicators and regulate the function of recipient cell (6). Tumor-derived EVs may act on different types of immune cells, such as effector T cells, nature regulatory T cells, natural killer cells, and macrophages, which contributes to the induction and maintenance of the immune-suppression environment (7, 8). Our previous data indicate that the EVs released from glioblastoma cells can transfer miR-214-5p to brain microglia/macrophages to modulate the corresponding inflammatory response that favors the survival of glioblastoma cells (9).

All of these indicate the potential of glioma in manipulating the immunosuppressive environment. This study was designed to decipher the potential role of GSC-derived EVs in microglia functions and the further detailed mechanism of the EV lncRNA metastasis-associated lung adenocarcinoma transcript 1 (MALAT1) (Supplementary Figure S1).

## Materials and Methods

### Patient Samples

Forty-five primary glioma tissues based on the WHO 2016 glioma classification were obtained during operation from the Second Hospital of Hebei Medical University. Written informed consent was obtained from the patients involved. The whole study procedure was approved by the Ethics Committee of the Second Hospital of Hebei Medical University (HMUSH-7223-o2).

### Cell Culture

Gentle MACS dissociators were utilized to dissociate and homogenize the glioma tissue into primary glioma tissue cells (GTCs), which were cultured in multipotent adult stem cell medium (60% DMEM low glucose, 40% MCDB-201, 2% fetal bovine serum, 10 ng/ml hPDGF, 10 ng/ml hEGF, 1 mg/ml linoleic acid–BSA, 10^−9^ M dexamethasone, 1 × ITS, and 10^−4^ M ascorbic acid-2 phosphate). After reaching 80% confluence, the GTCs were detached with trypsin and then stained with anti-CD24-FITC and anti-CD44-PE (Invitrogen, Carlsbad, CA, USA) antibodies (10 μg per 10^6^ cells). The CD44^+^CD24^−^ cells were considered to be GSCs (10). Microglia cells (HMC3, ATCC® CRL-3304™) were obtained from the American Type Culture Collection (ATCC, VA, USA), which were stimulated with lipopolysaccharide (LPS, 1 μg/ml) and cultured in RPMI-1640 medium (Gibco, Scoresby, VIC, Australia) supplemented with 10% FBS and 1% glutamine. All the cells were maintained in a humidified incubator (37°C, 5% CO_2_).

### EV Isolation

It is well-known that the technique adopted to isolate EVs is crucial in obtaining a homogenous population. In addition to ExoQuick-TC Exosome Precipitation, ultra-centrifuge was also utilized in this investigation to purify the EVs, which further strengthens and testifies the inflammatory modulation function of GSC-derived EVs. The conditioned medium from GSCs cells was firstly centrifuged to remove the cellular debris (10,000 × *g*, 10 min). Then, the commercially available kit ExoQuick-TC Exosome Precipitation Solution (System Biosciences, CA, USA) was adopted to isolate the EVs following the manufacturer's protocol. Briefly, conditioned medium and precipitation solution were mixed (1:1) and incubated (4°C, overnight), and then serially, centrifuges were performed (10,000 × *g* for 30 min; 140,000 × *g* for 5 min at 4°C) to get the GSC supernatant and the resultant EV pellet, which was re-suspended with 250–500 μl cell medium. The equal volume of EVs derived from GSCs was added to the microglia cells for 48 h of consecutive culture (9).

### Cell Transfection

si-MALAT1, miR-129-5p mimic, inhibitor, and normal control (NC) were manufactured by Genepharma Company (Shanghai, China) and transfected into GSCs and microglia cells at exponential phase (40–50% confluence, 50 nm) with Lipofectamine 2000 (Invitrogen, Carlsbad, CA, USA) on a six-well plate, which were further incubated for 48 h to extract RNA/protein. All of these performances were followed by the protocol recommended by the manufacturer.

### Cell-Counting Kit-8 Assay

Cell viability was detected using a Cell-Counting Kit-8 (CCK-8, Dojindo Laboratories, Tokyo, Japan). GSCs transfected with si-MALAT1, si-NC, or control cells (5 × 10^5^ cells) were plated into 96-well plates and cultured for 24 h. Then, 10 μl CCK-8 solution was added into each well and incubated for 2 h at 37°C. The absorbance at 450 nm was assayed with the SpectraMax Plus 384 Microplate Reader (Molecular Devices, MD, USA).

### 5-ethynyl-2'-deoxyuridine Staining

si-MALAT1 or si-NC transfected GSCs were seeded into a 96-well plate and cultured with 50 μmol/L 5-ethynyl-2′-deoxyuridine (EdU) (24 h, 37°C), which was further fixed with 3.75% paraformaldehyde for 10 min and penetrated with 0.5% Triton X-100 for 30 min. After that, 100 μl Click-iT® reaction cocktail was incubated with the cells for 30 min, and 5 μg/ml of Hoechst 33342 was utilized to stain the cell nuclei. Five random observation fields of each well were captured with a fluorescence microscope (Nikon 80i; Nikon, Tokyo, Japan).

### ELISA

The relative contents of interleukin (IL)-6, IL-8, and tumor necrosis factor (TNF)-α in the conditioned medium of microglia were assayed with commercially available ELISA kits (Abcam, La Jolla, CA, USA) following the manufacturer's instruction. The absorption (at 450 nm) was detected with the SpectraMax Plus 384 Microplate Reader (Molecular Devices, CA, USA), and the concentration was computed with a standard curve established with logic four-parameter fitting method.

### Real-Time PCR

Trizol reagent (Invitrogen, CA, USA) was utilized to extract total RNA from GSCs, GTCs, and microglia following the manufacturer's instruction. RNA (1 μg) was utilized to reversely transcribe with High-Capacity cDNA Reverse Transcription kits (Applied Biosystems, Foster City, CA, USA) and miScript Reverse Transcription Kit (Qiagen, Germantown, MD, USA). Quantitative PCR was performed with 2× FastStart Universal SYBR Green Master Mix (Roche Ltd., Basel, Switzerland) on ABI STEPONE with an initial denaturation of 95°C for 10 min, followed by 40 cycles of 95°C for 15 s and 60°C for 1 min. The relative mRNA expressions and MALAT1 expressions were calculated after being normalized to glyceraldehyde-3-phosphate dehydrogenase (GAPDH) or U6 expression. The primer sequences are listed in Table 1. Relative expression was quantified using the comparative ΔCT method.

**Table 1:** The sequence of primers (human) used for quantitative real-time PCR.

**Gene**	**Forward primer (5′-3′)**	**Reverse Primer (5′-3′)**
GAPDH	CAAGGTCATCCATGACAACTTTG	GTCCACCACCCTGTTGCTGTAG
U6	CTCGCTTCGGCAGCACA	AACGCTTCACGAATTTGCGT
MALAT1	AAAGCAAGGTCTCCCCACAAG	GGTCTGTGCTAGATCAAAAGGCA
HMGB1	TGCAGATGACAAGCAGCCTT	GCTGCATCAGGCTTTCCTTT
miR-129-5p	CGGCGGTTTTTTGCGGTCTGGGCT	CAACCTGGAGGACTCCATGCTG
IL-6	AATTCGGTACATCCTCGACGG	GGTTGTTTTCTGCCAGTGCC
IL-8	GACCACACTGCGCCAACAC	CTTCTCCACAACCCTCTGCAC
TNF-α	AGGCGCTCCCCAAGAAGACA	TCCTTGGCAAAACGCACCT
Nos2	TTCAGTATCACAACCTCAGCAAG	TGGACCTGCAAGTTAAAATCCC
Arg1	GTGGAAACTTGCATGGACAAC	AATCCTGGCACATCGGGAATC

*GAPDH, glyceraldehyde-3-phosphate dehydrogenase; MALAT, metastasis-associated lung adenocarcinoma transcript 11; HMGB1, high mobility group box-1 protein; IL, interleukin; TNF, tumor necrosis factor*.

### Western Blot

Protein (50 μg, in reducing conditions) was uploaded on 10% sodium dodecyl sulfate–polyacrylamide gel, which was further transferred onto a polyvinylidene difluoride membrane. The non-specific binding was blocked with 5% skim milk (0.05% TBST) for 1 h, and the membranes were subjected to a primary antibody specific for tumor susceptibility gene 101 (TSG101), apoptosis-linked gene 2 interacting protein X (Alix), CD63, high mobility group box-1 protein (HMGB1), and GAPDH (Santa Cruz Biotechnology Inc., Santa Cruz, CA) (4°C, overnight, 1:1,000 dilution). After washing with TBST, the bands were blotted with peroxidase-conjugated secondary antibody (room temperature, 1 h, 1:1,000 dilution) (Sigma-Aldrich, St. Louis, MO), and the signal was developed with an ECL system (GE Healthcare Life Sciences, Chalfont, UK). The densitometry was calculated with NIH-Image J (National Institutes of Health, Bethesda, MD, USA) by correcting for GAPDH.

### Luciferase Reporter Assay

TargetScan and miRcode databases were utilized to predict the target fragment regulated by miR-129-5p. MALAT1 wild type (WT), MALAT1 mutant (MUT), HMGB1 WT, and HMGB1 MU, which were predicted to bind with miR-129-5p, were subcloned into pGL4 luciferase reporter plasmid and co-transfected into microglial cells with miR-129-5p mimics or miR-NC. After 48 h of culture, the Dual-Luciferase Assay System (Promega, Madison, WI, USA) was used to measure the relative luciferase activities.

### Statistical Analysis

SPSS was used to analyze the data. The Student's *t*-test, one-way ANOVA followed by Tukey's test, or two-way ANOVA followed by the Bonferroni test was utilized to indicate the significance between different groups. Standard Spearman's rank correlation analysis was performed on the relative gene expression. The level of significance was set as *P* < 0.05.

## Results

### lncRNA MALAT1 Correlates With miR-129-5p and HMGB1 Expression in GSCs

**Figure 1:**
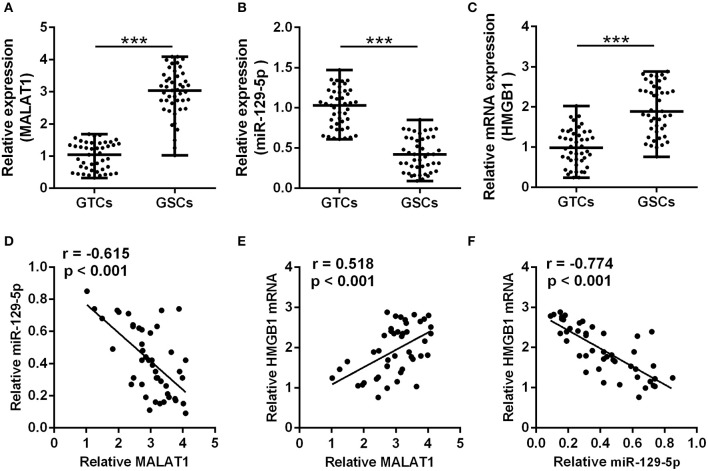
Correlation between metastasis-associated lung adenocarcinoma transcript 1 (MALAT1), miR-129-5p, and high mobility group box-1 protein (HMGB1) in glioma stem cells. **(A–C)** qRT-PCR was utilized to analyze the expressions of MALAT1 **(A)**, miR-129-5p **(B)**, and HMGB1 **(C)** between GSCs and GTCs from 45 GBM tissues. The data are presented as median with range. ***P < 0.001 between GTC and GSC group. **(D–F)** The expression correlations between MALAT1, miR-129-5p, and HMGB1 were analyzed with Spearman's correlation analysis. GTC, glioma tissue cell; GSC, glioma stem cell.

A total of 45 glioma patients were enrolled in this study, and the detailed demographics and pathology information are presented in Table 2. The GSCs and GTCs were further isolated, and the relative expressions of MALAT1 (Figure 1A), miR-129-5p (Figure 1B), and HMGB1 (Figure 1C) were detected. MALAT1 and HMGB1 expression in GSCs were remarkably higher than that of GTCs (*P* < 0.001), while miR-129-5 expression in GSCs was remarkably low compared with GTCs (*P* < 0.001). Spearman's rank correlation analysis in GSCs indicated that MALAT1 expression was negatively correlated with miR-129-5p expression (Figure 1D, *r* = −0.615, *P* < 0.001) and positively correlated with HMGB1 expression (Figure 1E, *r* = 0.518, *P* < 0.001), and it was further testified that HMGB1 expression was negatively correlated with miR-129-5p expression (Figure 1F, *r* = −0.774, *P* < 0.001).

**Table 2:** Patient characteristics.

**Variables**	***n* (%)**
Patients	45
Gender (m/f)	28/17
Age in year, median (range)	49.8 (19.3–81.7)
WHO grade	
II	6 (13.3%)
III	23 (51.1%)
IV	16 (35.6)
Tumor location	
Frontal	9 (20%)
Temporal	19 (42.2%)
Parietal	7 (15.6%)
Occipital	4 (8.9%)
Midline/basal ganglia/corpus callosum	6 (13.3%)
IDH1 R132H status	
Wild type	24 (53.3%)
Mutated	21 (46.7%)

### MALAT1 Promotes the Growth and Proliferation of GSCs

**Figure 2:**
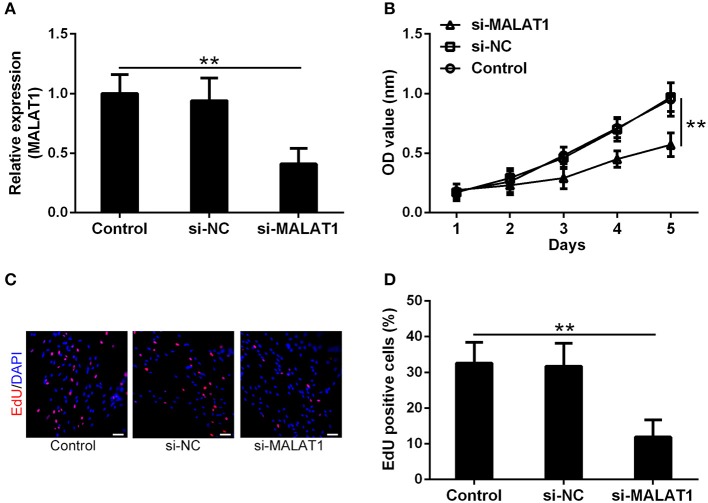
MALAT1 can promote glioma stem cell growth. **(A)** lncRNA MALAT1 expression in GSCs decreased significantly after si-MALAT1 transfection. **(B)** The growth of GSCs transfected with si-MALAT1 was inhibited significantly, indicated by the Cell-Counting Kit-8 (CCK-8) test. Representative 5-ethynyl-2′-deoxyuridine (EdU) staining **(C)** and the percentage of EdU-positive cells **(D)** among different groups. Data were presented as mean ± SD from three independent experiments. **P < 0.01 between the indicated groups.

To testify the function of MALAT1 involved in GSCs, si-MALAT1 and si-NC transfected GSCs systems were established. As shown in Figure 2A, more than 50% reduction of MALAT1 was achieved in the si-MALAT1 interfering group compared with the control and si-NC groups (*P* < 0.01), which indicated the successful establishment of MALAT1 interfering GSCs. Cell viability indicated by CCK-8 assay showed that si-MALAT1 could significantly suppress GSC growth when compared with the si-NC group and control group (Figure 2B, *P* < 0.01). At the same time, EdU-positive cells in the si-MALAT1 group declined significantly when compared with control and si-NC groups, which suggested that MALAT1 interfering repressed GSCs proliferation (Figures 2C,D, *P* < 0.01). All of these indicated that MALAT1 could promote GSC growth and proliferation.

### GSC-Derived EV MALAT1 Modulates the LPS-Induced Inflammatory Response of Microglia

**Figure 3:**
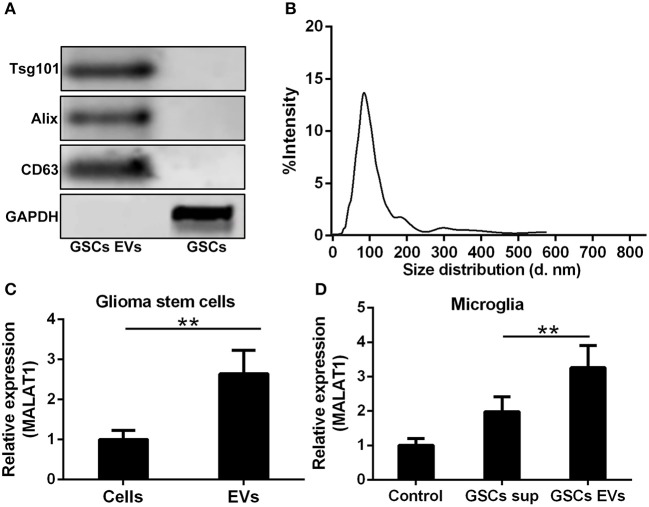
Highly expressed MALAT1 in the extracellular vesicles (EVs) of GSCs. **(A)** Western blot characterization of GSC EVs with EV marker antibody against Tsg101, Alix, and CD63. **(B)** The size distribution of EVs from glioma stem cells. **(C)** Highly expressed MALAT1 in the EVs of GSCs. **(D)** qRT-PCR analysis of MALAT1 after microglia were exposed to glioma stem cell supernatant (GSC sup), EVs from glioma stem cells (GSC EVs), or pure media (Control) for 48 h. The relative MALAT1 expressions were calculated after being normalized to U6 expression. Data were presented as mean ± SD from three independent experiments. **P < 0.01 between the indicated groups.

Whether the abundance of MALAT1 in GSCs was packaged into EVs and secreted into the extracellular microenvironment as the intercellular messenger was determined in this investigation. GSC-derived EVs were isolated and characterized by the detection of EV specific markers, such as Tsg101, Alix, and CD63 (Figure 3A). It is worth noting that in reducing conditions, CD63 exhibited a clear band at 26 kDa corresponding to unprocessed CD63 together with another band at 63 kDa, which represented a glycosylated form. The size distribution of EVs derived from GSCs is shown in Figure 3B, which indicated that the isolated EVs were uniform in size. MALAT1 was significantly enriched in GSC-derived EVs compared with GSCs (Figure 3C, *P* < 0.01). Application of GSC-derived EVs and GSC supernatant (GSC sup) could remarkably increase the intracellular contents of MALAT1 in cultured microglia, and the relative expression of MALAT1 in microglia was significantly higher in the GSC-derived EVs group compared with the GSC supernatant group (1.5-fold, *P* < 0.01, Figure 3D), which indicated that EVs derived from GSCs could promote the accumulation of MALAT1 in microglia.

**Figure 4:**
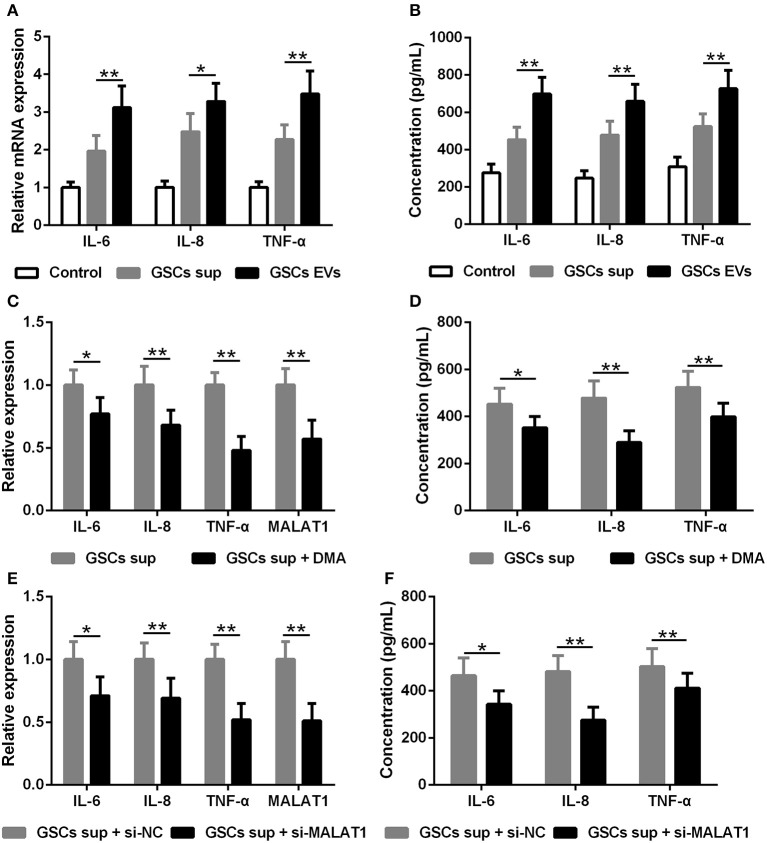
EV lncRNA MALAT1 released from glioma stem cells modulates the inflammatory response of microglia after lipopolysaccharide stimulation. **(A)** qRT-PCR assay of interleukin (IL)-6, IL-8, and tumor necrosis factor (TNF)-α in microglia exposed to GSC sup, GSC EVs, or pure media (Control) for 48 h after LPS stimulation. **(B)** ELISA tested the concentration of IL-6, IL-8, and TNF-α in microglia cells exposed to GSC sup, GSC EVs, or pure media (Control) for 48 h after LPS stimulation. **(C)** qRT-PCR assay of MALAT1, IL-6, IL-8, and TNF-α in microglia exposed to GSC sup and GSC sup plus dimethyl amiloride (GSC sup + DMA) for 48 h after LPS stimulation. **(D)** The concentration of IL-6, IL-8, and TNF-α in microglia exposed to GSC sup and GSC sup + DMA for 48 h after LPS stimulation. **(E)** Forty-eight hours after glioma stem cells were transfected with siMALAT1 or negative control si-NC, a qRT-PCR assay of MALAT1, IL-6, IL-8, and TNF-α in microglia exposed to the resulting culture supernatant for 48 h after LPS stimulation. **(F)** Forty-eight hours after glioma stem cells were transfected with siMALAT1 or negative control si-NC, ELISA analysis of IL-6, IL-8, and TNF-α concentration in microglia exposed to the resulting culture supernatant for 48 h after LPS stimulation. The relative mRNA expressions and MALAT1 expressions were calculated after being normalized to GAPDH or U6 expression. Data were presented as mean ± SD from three independent experiments. *P < 0.05, **P < 0.01 between the indicated groups.

The impact of EV MALAT1 on the inflammatory response of microglia challenged by LPS was evaluated. The relative mRNA expression of IL-6, IL-8, and TNF-α in LPS-stimulated microglia was simultaneously induced by conditioned medium from GSCs and GSC-derived EVs (Figure 4A). Consistently, the concentration of IL-6, IL-8, and TNF-α in the microglia culture medium increased as indicated by ELISA assay (Figure 4B). Pre-treated with dimethyl amiloride (DMA) for 2 h, EV releasing inhibitor and EV pathway blocker could diminish the up-regulated expression and secretion of IL-6, IL-8, and TNF-α cultured with GSC supernatant (Figures 4C,D), and the expression of MALAT1 in microglia was also down-regulated by DMA administration (Figure 4C), which indicated that the accumulation of MALAT1 in microglia could be down-regulated by EV releasing inhibition. Furthermore, the supernatant derived from si-MALAT1 interfering GSCs, which could diminish the EV transfer of MALAT1 from GSCs to microglia, down-regulated the expression (Figure 4E) and secretion (Figure 4F) of IL-6, IL-8, and TNF-α when compared with the supernatant derived from si-NC interfering GSCs.

### MALAT1 Inhibits miR-129-5p to Up-Regulate HMGB1 Expression in Microglia Cells

**Figure 5:**
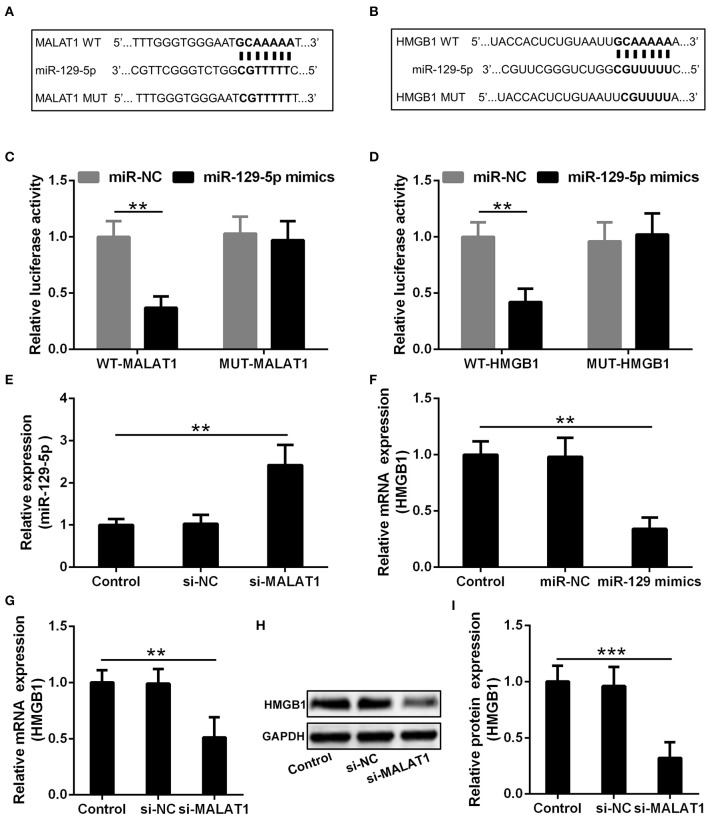
MALAT1 inhibits miR-129-5p to regulate the expression of HMGB1 in microglia cells. **(A)** The suspected binding sites of miR-129-5p with the wild type of MALAT1 are shown. MALAT1 fragment with mutated binding site with miR-129-5p is also indicated. **(B)** The suspected binding sites of miR-129-5p with the wild type of HMGB1 are shown. A mutated miR-129-5p binding sequence of HMGB1 is also shown. **(C)** Luciferase reporter plasmids containing wild-type MALAT1, mutated MALAT1, were co-transfected with miR-129-5p mimics or miR-NC into microglial cells. The firefly luciferase reporter activities were normalized with Renilla luciferase reporter activities. **(D)** Luciferase reporter plasmids containing wild-type HMGB1, mutated HMGB1, were co-transfected with miR-129-5p mimics or miR-NC into microglia cells. The firefly luciferase reporter activities were normalized with Renilla luciferase reporter activities. **(E)** The relative expression of miR-129-5p in microglia cells transfected with si-MALAT1 or si-NC was analyzed by qRT-PCR. **(F)** The mRNA expression of HMGB1 in microglia transfected with miR-129-5p mimics or miR-NC was analyzed by qRT-PCR. **(G–I)** The relative mRNA and protein expression of HMGB1 in microglia cells transfected with si-MALAT1 or si-NC were analyzed by qRT-PCR and Western blotting. Data were presented as mean ± SD from three independent experiments. **P < 0.01, ***P < 0.001 between the indicated groups.

The potential binding site of MALAT1 and miR-129-5p revealed by the miRcode database is presented in Figure 5A, and the predicted binding site between miR-129-5p and HMGB1 indicated by the TargetScan database is displayed in Figure 5B. In order to testify the realness of specific binding and further transcriptional activity, luciferase reporter vectors containing MALAT1 WT, MALAT1 MUT, HMGB1 WT, and HMGB1 MUT were constructed. Luciferase reporter assay showed that miR-129-5p could restrain the luciferase activity of the reporting vector containing the MALAT1 sequence significantly (Figure 5C, *P* < 0.01), and miR-129-5p could inhibit the luciferase activity of the reporting vector containing the HMGB1 sequence significantly in microglia cells (Figure 5D, *P* < 0.01). It was further demonstrated that microglia cells transfected with si-MALAT1 showed increased miR-129-5p expression compared with the si-NC group (Figure 5E, *P* < 0.01), and microglia cells transfected with miR-129-5p mimics could down-regulate the expression of HMGB1 compared with the miR-NC group (Figure 5F, *P* < 0.01). On the other hand, microglia cells transfected with si-MALAT1 could down-regulate the mRNA (Figure 5G, *P* < 0.01) and protein (Figures 5H,I, *P* < 0.001) expression of HMGB1 when compared with the si-NC group. All of these indicated that MALAT1 could inhibit miR-129-5p to regulate HMGB1 expression in microglia cells.

### EV lncRNA MALAT1 Released From GSCs Modulates the Inflammatory Response of Microglia After LPS Stimulation Through the miR-129-5p/HMGB1 Axis

**Figure 6:**
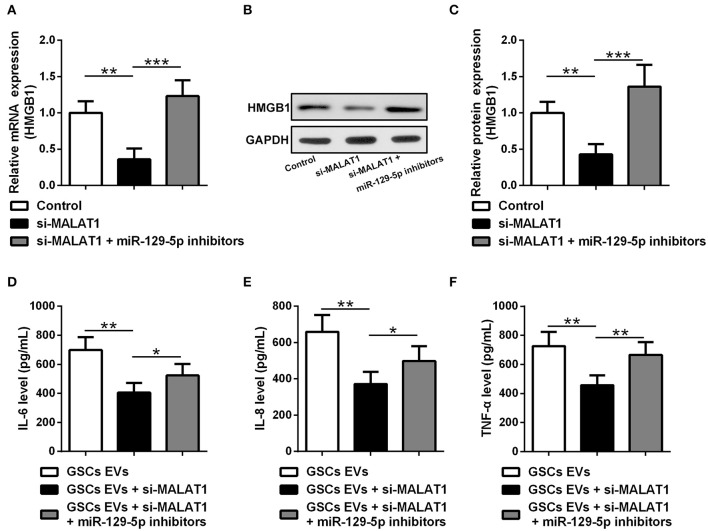
EV MALAT1 released from glioma stem cells modulates the inflammatory response of microglia after lipopolysaccharide stimulation through the miR-129-5p/HMGB1 axis. **(A–C)** The relative mRNA and protein expressions of HMGB1 in microglia cells transfected with si-MALAT1 or si-MALAT1 plus miR-129-5p inhibitors were analyzed by qRT-PCR and Western blotting. **(D–F)** GSCs were transfected with siMALAT1 for 48 h, and then EVs were isolated. Microglia cells transfected with miR-129-5p inhibitors for 48 h. The concentration of IL-6, IL-8, and TNF-α in microglia exposed to EVs from glioma stem cells (GSC EVs), GSCs + siMALAT1, or GSCs + siMALAT1 + miR-129-5p inhibitors for 48 h after LPS stimulation. Data were presented as mean ± SD from three independent experiments. *P < 0.05, **P < 0.01, ***P < 0.001 between the indicated groups.

HMGB1 mRNA (Figure 6A) and protein (Figures 6B,C) expression were down-regulated by si-MALAT1 treatment (*P* < 0.01), and miR-129-5p inhibitors could rescue such decrease (*P* < 0.001). To further evaluate the regulatory effect of GSC EV-derived MALAT on miR-129-5p and HMGB1 expression in LPS-stimulated microglia, miR-129-5p inhibitor transfected microglia were co-cultured with GSC EVs, which were isolated after GSCs were transfected with siMALAT1 and cultured for 48 h. The supernatant detected with ELISA revealed that the secretion of IL-6 (Figure 6D), IL-8 (Figure 6E), and TNF-α (Figure 6F) in microglia was significant down-regulated by EVs derived from si-MALAT1 transfected GSCs (*P* < 0.01), and such effects could be restored by the transfection of miR-129-5p inhibitor in microglia (*P* < 0.05 or *P* < 0.01). It was worth noting that EV-derived MALAT1 released from GSCs could promote the polarization of LPS-stimulated microglia into the M2 phenotype (up-regulated Nos2, Supplementary Figure S2). All of these findings indicated that EV-derived MALAT1 could modulate the inflammatory response of microglia after LPS stimulation through regulating the miR-129-5p/HMGB1 axis *in vitro*.

## Discussion

In this investigation, both the GSCs isolated from low-grade glioma and high-grade glioma can modulate the inflammatory response of microglia after LPS stimulation. It is further testified that lncRNA MALAT1 is transferred from GSC cells to surrounding microglia via EV secretion, which consequently modulates inflammatory response through the miR-129-5p/HMGB1 axis to influence the secretion of IL-6, IL-8, and TNF-α.

Tumor microenvironment and the interactions between glioma cells and other origins of cells are vital for glioma development and progress; for a low incidence of metastasis, glioma can be considered as a localized solid tumor. Previous investigations are focused on the cytokine- and chemokine-mediated intercellular communications between glioma cells and immune cells. Recently, EV relevant nucleotide transfer is increasingly recognized to play a cardinal function in the establishment of a supportive and immunosuppressive environment for glioma survival and aggressive invasion. Metastasis-associated MALAT1 is a favorable prognostic factor for lung cancer, colorectal cancer, bladder cancer, and glioma (11–13). It is also indicated that the aberrant overexpression of MALAT1 is associated with WHO glioma grade significantly (I–II vs. III–IV; *P* = 0.007) and with glioma size (<3 cm vs. *T* ≥ 3 cm; *P* = 0.008). This investigation is consistent with the previous report that MALAT1 expression is up-regulated significantly in GSCs compared with tumor cells, and miR-129-5p is down-regulated in GSCs (10). Notably, MALAT1 is greatly enriched in the secreted EVs from the cultured GSCs, which indicates the highly selective package of MALAT1 in the EVs.

MicroRNAs could be sponged and regulated by lincRNA MALAT1 (14). It is testified in this research that MALAT1 can function as a competing endogenous ceRNA to up-regulate HMGB1 expression by sponging miR-129-5p in microglia upon LPS stimulation. Cumulative evidence has indicated the context-dependent roles of HMGB1, which can protect microglia from injury and inflammatory stimulation. Consequently, it is testified in this research that GSCs could induce microglia to produce cytokines for their own survival through MALAT1 dependent EV-mediated cellular interaction. Elevated expression of IL-6 is inversely correlated with the survival time of glioma patients, and IL-6 is involved in glioma growth, angiogenesis, and resistance to chemotherapy, radiation, and apoptosis (15–17). Besides, IL-8 is critical to glial tumor neovascularity and progression, which correlates with the histologic grade in glioblastoma (18). TNF-α is testified to induce glioma cell invasion (19). All of these suggest that MALAT1 dependent EV-mediated microglia stimulation and IL-6, IL-8, and TNF-α secretion could contribute to glioma progression (20).

The systemic immunomodulatory character of EVs is recognized in neuro-oncology. Glioblastoma multiforme–derived EVs could affect the cytokine secretion, and the migratory ability of healthy peripheral blood mononuclear cells (PBMCs) stimulated with mitogen (21) skews the differentiation of PBMCs into tumor-supportive macrophages (22) and boosts a Th2 type inflammation environment (23, 24). All of these indicate that glioma can exert a systemic immunosuppressive function beyond the boundaries of the CNS.

On the other hand, the technique adopted to isolate EVs is crucial in obtaining a homogenous population and reliable downstream clinical data (25). As EVs derived from GSCs represent a mediator for intercellular communication, they could be designed as a tool to reprogram anti-tumor immunity (26). Nucleotide chemistry and nanoparticle technology have been advanced to improve lincRNA stability and cell penetration ability, and immune-targeting has also been designed to promote the specificity of lincRNA delivery. Although not all the aspects of MALAT1 derived from EVs in tumor inflammation have been deciphered, MALAT1 derived from GSC EVs could represent a specific target to treat glioma (27).

## Conclusions

Our study highlights the role of EV MALAT1-miR-129-5p-HMGB1 in the modulation of the inflammatory response in microglial cells, which holds great promise to serve as a therapeutic target for glioma treatments.

## Data Availability Statement

The datasets generated for this study are available on request to the corresponding author.

## Ethics Statement

The studies involving human participants were reviewed and approved by the Ethics Committee of The Second Hospital of Hebei Medical University. The patients/participants provided their written informed consent to participate in this study.

## Author Contributions

JiaY, GS, YH, JipY, YS, HongjL, CL, YW, ZL, JN, HonglL, XS, HW, PL, and BJ: did the experiments. JiaY, GS, JipY, YS, HongjL, CL, YW, ZL, JN, and HonglL: analyzed the data. JiaY, GS, HW, PL, and BJ: conceived this study. JiaY: wrote the manuscript.

### Conflict of Interest

The authors declare that the research was conducted in the absence of any commercial or financial relationships that could be construed as a potential conflict of interest.

## Abbreviations

GSCs: glioma stem cells
GTCs: glioma tissue cells
LPS: lipopolysaccharide
HMGB1: high mobility group box-1 protein
TNF: tumor necrosis factor.
