# Supplementary material for: Extracellular Vesicle lncRNA Metastasis-Associated Lung Adenocarcinoma Transcript 1 Released From Glioma Stem Cells Modulates the Inflammatory Response of Microglia After Lipopolysaccharide Stimulation Through Regulating miR-129-5p/High Mobility Group Box-1 Protein Axis
Source: Front Immunol. 2020 Feb 7;10:3161. doi: 10.3389/fimmu.2019.03161 (PMC7020807; doi:10.3389/fimmu.2019.03161)
Supplement: Supplementary file 1 [file Data_Sheet_1.PDF]

Supplementary Materials

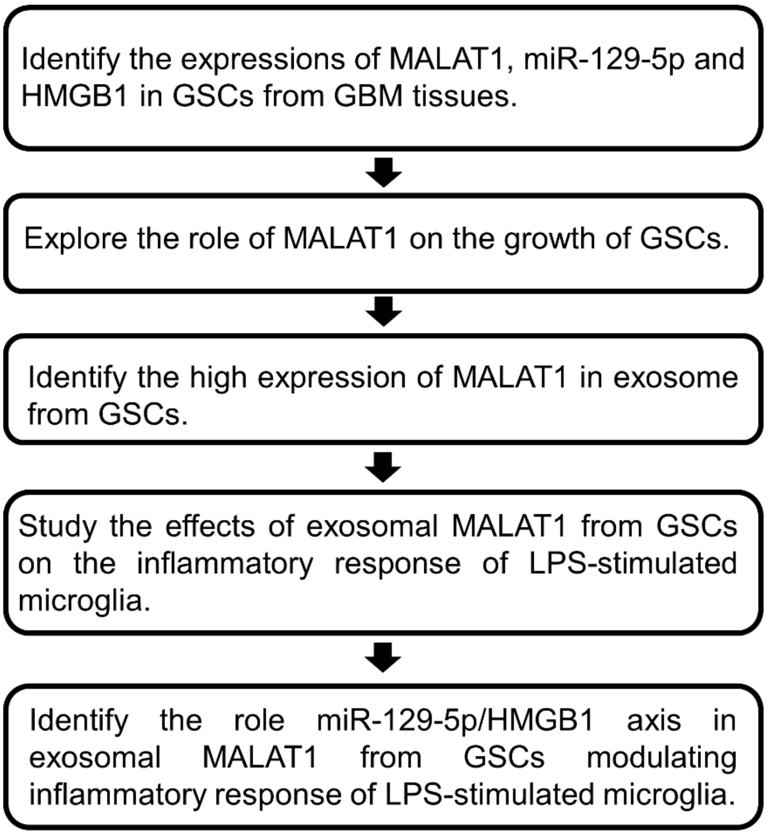

Figure S1. Schematic of the study.

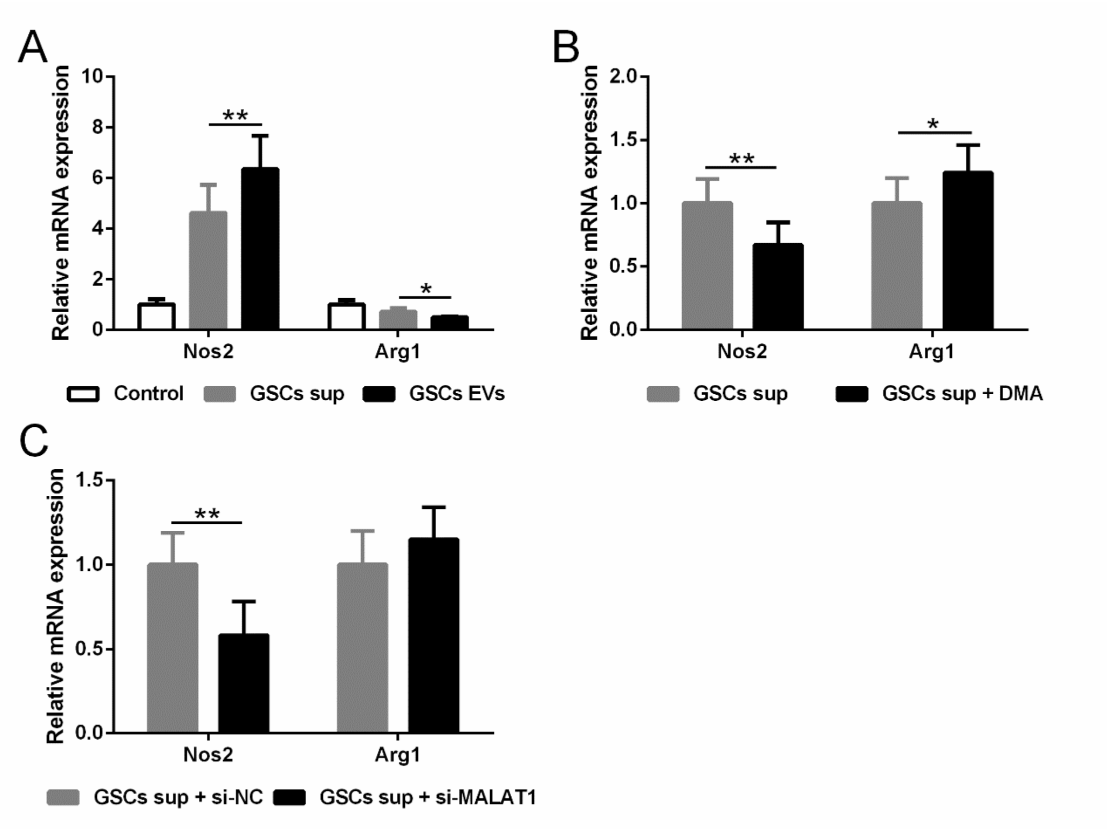

Figure S2. EVs lncRNA MALAT1 released from glioma stem cells modulates the

M1/M2 phenotype of microglia after lipopolysaccharide stimulation. A) qRT-PCR assay of Nos2 and Arg1 in microglia exposing to GSCs supernatant (GSCs sup), EVs from GSCs (GSCs EVs) or pure media (Control) for 48 h after LPS stimulation. B) qRT-PCR assay of Nos2 and Arg1 in microglia exposing to glioma stem cells supernatant (GSCs sup) and GSCs sup plus DMA (GSCs sup + DMA) for 48 h after LPS stimulation. C) 48 h after glioma stem cells were transfected with siMALAT1 or negative control si-NC, qRT-PCR assay of Nos2 and Arg1 in microglia exposing to exposing to the resulting culture supernatant for 48 h after LPS stimulation.
